# TCR signaling by conventional CD4^+^ T cells is required for optimal maintenance of peripheral regulatory T cell numbers

**DOI:** 10.1002/iid3.100

**Published:** 2016-03-24

**Authors:** Theresa M. Leichner, Atsushi Satake, Taku Kambayashi

**Affiliations:** ^1^ Department of Pathology and Laboratory MedicinePerelman School of Medicine at the University of PennsylvaniaPhiladelphiaPA19104USA; ^2^ First Department of Internal MedicineKansai Medical UniversityMoriguchiOsakaJapan

**Keywords:** Homeostasis, IL‐2, indexing, regulatory T cell

## Abstract

To maintain immune tolerance, regulatory T cell (Treg) numbers must be closely indexed to the number of conventional T cells (Tconvs) so that an adequate Treg:Tconv ratio can be maintained. Two factors important in this process are the cytokine interleukin‐2 (IL‐2) and T cell receptor (TCR) stimulation by major histocompatibility complex class II (MHC‐II). Here, we report that in addition to TCR stimulation of Tregs themselves, the maintenance of Tregs also requires TCR signaling by Tconvs. We found that Tconvs produce IL‐2 in response to self‐peptide‐MHC‐II complexes and that Tconvs possessing more highly self‐reactive TCRs express more IL‐2 at baseline. Furthermore, selective disruption of TCR signaling in Tconvs led to a trend toward decreased expression of IL‐2 and attenuated their ability to maintain Treg numbers. These data suggest that in order to maintain an adequate Treg:Tconv ratio, Tregs are continuously indexed to self‐peptide‐MHC‐II‐induced TCR signaling of Tconvs. These results have implications in attempts to modulate immune tolerance, as Treg numbers adjust to the self‐reactivity, and ultimately IL‐2 production by the T cells around them.

## Introduction

Development of immunological tolerance to self is an essential biologic process to prevent over‐activation of the immune system and resulting autoimmunity. This tolerance is effectively achieved through selection of T lymphocytes with low affinity to self‐antigens as well as the creation and maintenance of Foxp3^+^ regulatory T cells (Tregs) a subset of T cells with suppressive function. Failure to develop Tregs results in the development of a widespread, fatal autoimmune disease 1, 2. The maintenance of the peripheral Treg population is achieved through a combination of survival and proliferation attained by signaling through the cytokine receptor for interleukin 2 (IL‐2) and through the T cell receptor (TCR) 3, 4, 5, 6.

Despite the homeostatic requirement for IL‐2 and the expression of the high affinity IL‐2 receptor (CD25) on Tregs, these cells do not produce this cytokine themselves 4. Instead, Treg maintenance and/or development depends on IL‐2 produced by other TCRαβ^+^ T cells 7. In fact, it has been shown that the number of Tregs surviving in the periphery is directly indexed to the number of CD4^+^ conventional T cells (Tconvs) able to produce IL‐2 7. However, the mechanism by which IL‐2 is produced by Tconvs in the steady state to maintain Tregs is not known. As TCR stimulation drives IL‐2 production in T cells, we hypothesized that Treg numbers might be indexed to TCR signaling by Tconvs (as a readout of activation state), rather than to the absolute numbers of Tconvs.

To test this notion, we hereby examined the role of TCR signaling in Tconvs at the steady state for both their ability to produce IL‐2 as well as their capacity to maintain the Treg population. Through in vivo and in vitro approaches, we find that Tconvs produce IL‐2 through sub‐activating TCR stimulation by self‐peptide MHC class II (MHC‐II) complexes. Furthermore, the selective attenuation of TCR signaling in Tconvs results in decreased IL‐2 production and an impairment in Treg maintenance. Our data suggest that Treg numbers are indexed to TCR signaling by Tconvs, both from sub‐threshold self‐antigens in the steady state as well as foreign, activating antigens in an immune response.

## Materials and Methods

**Mice**

Y145F knock‐in mice, SLP‐76^flox/Y145F^ conditional mutant (cY145F), and SLP‐76^flox/+^ conditional heterozygous (cSLP76) mice were generated as previously described 8, 12 and bred in our facility. TCRβ/δ KO, I‐Abβ KO (MHC‐II KO), C57BL/6 CD90.1, C57BL/6 Foxp3.GFP and C57BL/6.SJL Foxp3.GFP reporter mice were purchased from The Jackson Laboratory (Bar Harbor, ME) or Charles River (Kingston, NY) and were bred and maintained in our animal facility. Mice were housed in pathogen‐free conditions and treated in strict compliance with Institutional Animal Care and Use Committee regulations of the University of Pennsylvania.

### Flow cytometry, cell sorting, and data analysis

Antibodies for flow cytometry were purchased from eBioscience (San Diego, CA), BD Bioscience (San Jose, CA), or Tonbo Bioscience (San Diego, CA). Flow cytometry was performed with an LSR II, FACSCanto, or a FACSCalibur. Cell sorting was performed with a FACSAria cell sorter (BD Biosciences) or MACS Cell Separation (Miltenyi Biotec, San Diego, CA). Data were analyzed with FlowJo software (TreeStar) and Prism (GraphPad).

### In vitro co‐cultures and IL‐2 detection

FACS‐sorted Naïve Tconvs (CD4^+^CD25^−^CD45RB^hi^) from either C57BL/6 or SLP76.Y145F mice were co‐cultured with CD11c^+^ MACS‐sorted DCs at a 1:1 ratio in 200 µL T cell media (MEM‐α with 10% FBS, 1% penicillin/streptomycin, 10 mM HEPES, and 1 × 10^−5^ M 2‐mercaptoethanol) with mouse GM‐CSF (10 ng/mL; PeproTech, Rocky Hill, NJ). Culture supernatant was collected at 96 h and analyzed by ELISA for IL‐2 production. CD11c^+^ DCs were sorted from spleens of mice subcutaneously injected 8–10 days prior with FLT3L‐expressing EL4 cells. IL‐2 was detected using the Mouse IL‐2 ELISA Ready‐SET‐Go! kit (eBioscience).

### CD5 high and low Tconv sort, RNA extraction, and Quantitative PCR

Tconvs were FACS sorted using a FACSAria sorting on CD4^+^CD25^−^ T cells and then on CD5 levels (highest and lowest 20%). RNA was isolated using QIAshredder columns paired with the RNeasy minikit (QIAGEN; Germantown, MD). Expression of IL‐2 mRNA was measured by real‐time PCR (Applied Biosystems StepOnePlus Real‐Time PCR System; Carlsbad, CA) using SYBR Green Master Mix (Applied Biosystems) on 1000‐cell equivalents of cDNA template and 100 nM primer concentration. The oligonucleotides used to amplify the template DNA were *Il2* fwd, 5′‐AGCAGCTGTTGATGGACCTA‐3′; *Il2* rev, 5′‐CGCAGAGGTCCAAGTTCAT‐3′; *18S* fwd, 5′‐TCAAGAACGAAAGTCGGAGG‐3′; *18S* rev, 5′‐GGACATCTAAGGGCATCACA‐3′.

### Irradiation, Reconstitution and IL‐2 IC treatment

C57BL/6.SJL mice were lethally irradiated with a split dose of 11 Gy and reconstituted with 5 × 10^6^ MACS‐purified T cell‐depleted (CD90.2) bone marrow of either C57BL/6 or MHC‐II KO origin. At the same time as the bone marrow transfer, the mice received 5 × 10^6^ MACS‐purified CD4^+^ T cells. At day 14 post‐transfer, mice were treated with IL‐2 immune complexes (0.25 μg IL‐2 and 1.25 μg αIL‐2 mAB) or PBS for 5 days. Treg percentages were measured in the peripheral blood at day 14, 20, and 27 post‐transfer.

### Adoptive transfers and Tamoxifen administration

Tconvs (CD45.2^+^CD4^+^CD25^−^) were FACS‐sorted from spleens of SLP‐76^flox/Y145F^ conditional mutant (cY145F) and SLP‐76^flox/+^ conditional heterozygous (cSLP76) mice. Tconvs from either source were transferred in a 4:1 ratio with FACS‐sorted WT Tregs (CD45.1^+^CD4^+^GFP^+^) from C57BL/6.SJL Foxp3.GFP reporter mice into TCRβ/δ KO mice. For deletion of the loxp‐flanked SLP‐76 allele 8–10 weeks after cell transfer, mice were orally given 200 µg/g body weight of Tamoxifen in corn oil every day for 5 days. Mice were bled weekly to measure circulating Tconvs and Tregs for 12 weeks. Spleens were dissociated and set in erythrocyte lysis buffer (140 mM NH_4_Cl, 17 mM Tris pH 7.5) for 2 min. Cells were then filtered through 70 µm nylon mesh to obtain a single cell suspension for flow cytometry staining. Treg percentages were assessed as CD4^+^CD45.1^+^Foxp3^+^ percent of total CD4^+^ T cells.

## Results and Discussion

### Tconvs produce IL‐2 in response to self peptide‐MHC‐II complexes

**Figure 1:**
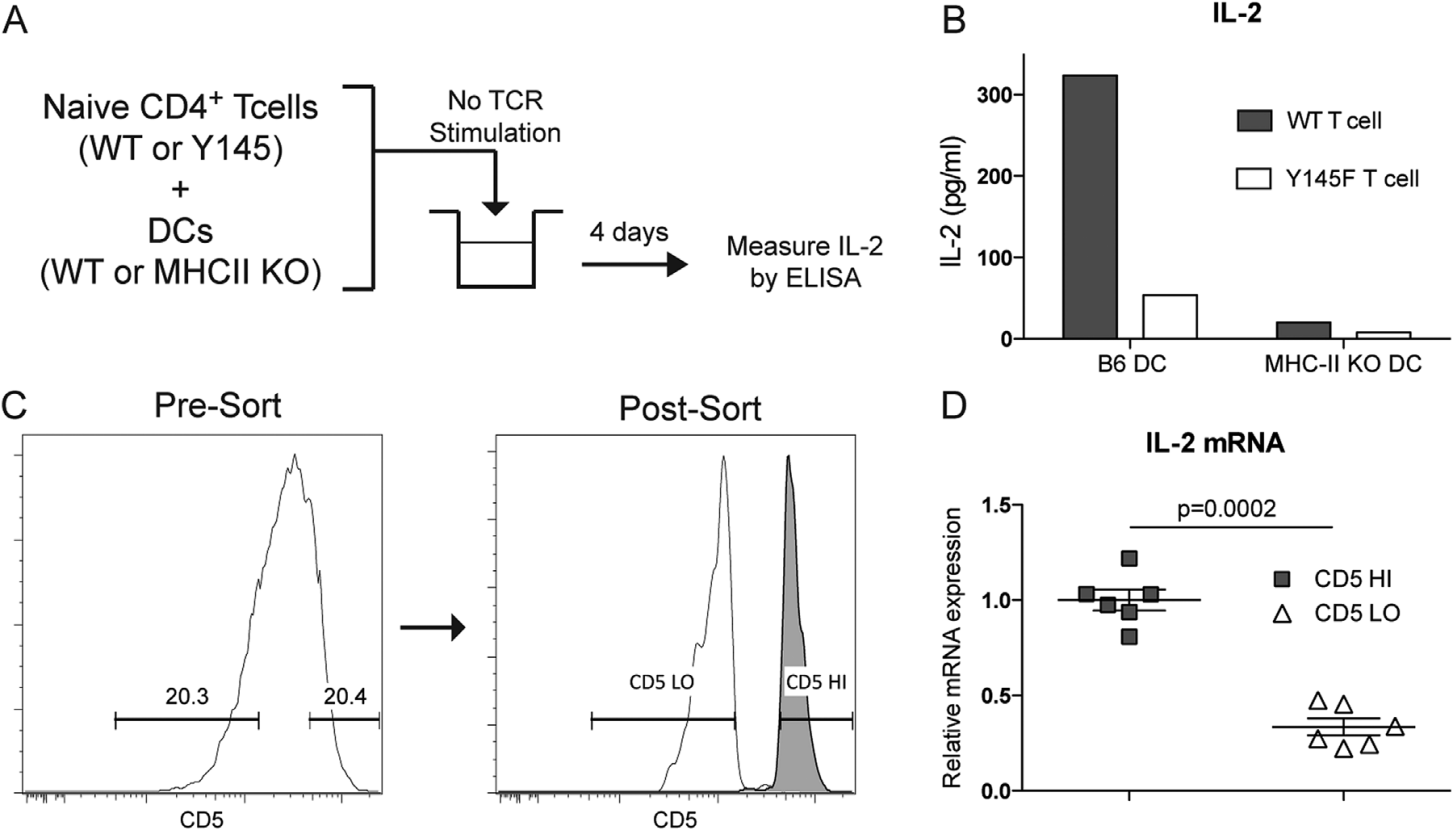
IL‐2 is induced by stimulation of CD4^+^ Tconvs by self peptide‐MHC‐II complexes (A). Naïve T cells (CD4^+^CD25^−^CD45RB^hi^) from WT or Y145F mice were FACS‐sorted and co‐cultured at a 1:1 ratio with DCs from either WT or MHC‐II KO mice with no added TCR stimulation. (B) Ninety‐six hours later, IL‐2 content in the supernatant was assessed by ELISA. One representative of two experiments is shown. (C) Sorting strategy for upper and lower 20% of CD5 expressing (CD5^hi^ and CD5^lo^, respectively) Tconvs (CD4^+^GFP^−^) cells from C57BL/6 Foxp3.GFP reporter mice is shown. (D) IL‐2 mRNA expression in the CD5^hi^ and CD5^lo^ Tconv populations, plotted as mean ± SEM of six mice from two individual experiments is shown. Statistical analysis was performed using two‐tailed paired Student's *t*‐test.

We hypothesized that Tconvs produce IL‐2 in the steady state due to interactions of their TCR with self‐peptide MHC‐II complexes. To test this hypothesis, we first tested the ability of self‐peptide MHC‐II complexes to stimulate TCR‐mediated IL‐2 production in an in vitro system (Fig. 1A). When co‐cultured with syngeneic DCs, naïve WT Tconvs (CD4^+^CD45RB^hi^CD25^−^) produced IL‐2 in response to syngeneic WT DCs but not when the DCs were derived from MHC‐II KO mice (Fig. 1B). Next, we disrupted TCR signaling in response to MHC‐II ligation by using T cells from mice with a Y→F mutation in Y145 (Y145F) of the adaptor molecule SLP‐76, which leads to decreased TCR‐mediated PLCγ1 activation 8. Co‐culture of naive Y145F Tconvs with syngeneic DCs showed significantly decreased IL‐2 production compared to WT Tconvs (Fig. 1B). Together, these data suggest that self‐peptide MHC‐II complexes induce IL‐2 production in a TCR/MHC‐II signaling‐dependent manner.

To test the role of TCR/self MHC‐II peptide complex interactions in IL‐2 production in vivo, T cells possessing high affinity TCRs were compared to T cells with low affinity TCRs against self MHC‐II peptide complexes. The expression level of CD5 on T cells correlates with TCR affinity to self MHC‐II peptide complexes, which is established during thymic selection and maintained in the periphery 9. Recent work has shown that Tconvs with higher affinity for self‐peptide MHC‐II, as detected by the amount of CD5 expression, have a greater level of proximal TCR signals in the form of TCR ζ‐chain phosphorylation 10. Consistent with our hypothesis, we have found that Tconvs with high CD5 expression (top 20%) have a significantly elevated amount of IL‐2 mRNA expression in comparison to Tconvs with low CD5 expression (bottom 20%; Fig. 1C and D). This suggests that steady‐state IL‐2 production by Tconvs correlates with their TCR affinity for self‐peptide MHC‐II complexes.

### TCR signaling by Tconvs is required for maintenance of the Tconv:Treg ratio

**Figure 2:**
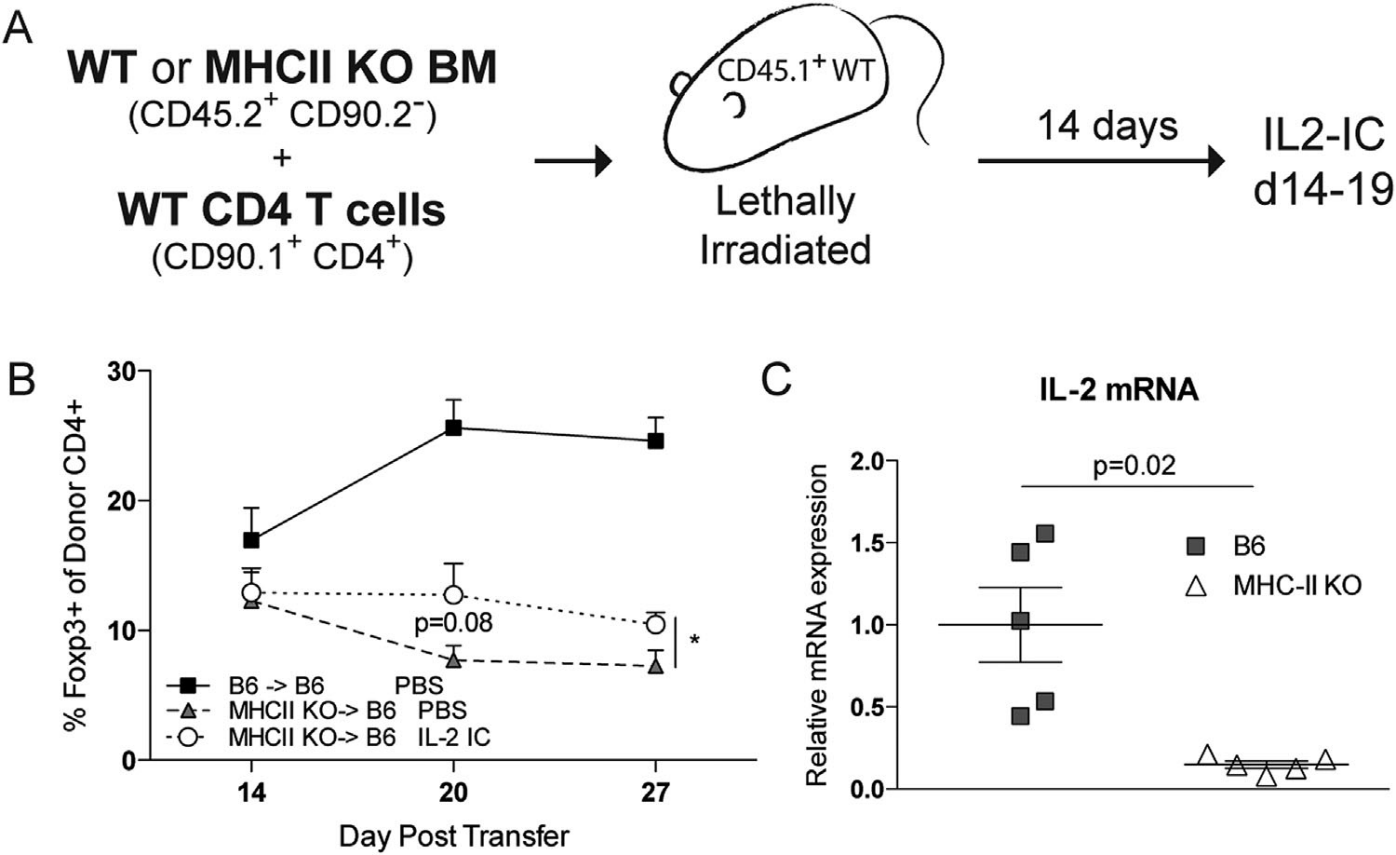
Lack of IL‐2 production in MHCII deficient environment leads to decreased Treg percentages (A). T cell depleted bone marrow from WT or MHCII KO mice (both CD45.2^+^) was acquired through MACS‐sorting and transferred with MACS‐sorted CD4^+^ T cells (CD90.1^+^) into lethally irradiated WT (CD45.1^+^) hosts. IL‐2 IC (0.25 μg IL‐2 and 1.25 μg αIL‐2 mAB) or PBS control were given from day 14–19. (B) Treg percentages were measured in the peripheral blood over time after cell transfer to irradiated recipients. Summary data plotted as the mean ± SEM (*n* = 12 per group) from three independent experiments. (C) IL‐2 mRNA was measured from the CD90.1^+^CD4^+^CD25^−^ FACS‐sorted Tconvs from the spleens of WT or MHCII KO chimeras at day 21 post‐transfer. Summary data plotted as the mean ± SEM (*n* = 5 per group) of 2 independent experiments. *p < 0.05 or as noted by unpaired, two tailed Student's *t*‐test.

To test if the baseline TCR interaction with self‐peptide MHC‐II complexes was important in maintaining Treg numbers, we utilized an adoptive transfer system in which expression of MHC‐II was lacking in hematopoietic cells. In this model, lethally irradiated CD45.1^+^ WT hosts were reconstituted with CD45.2^+^ WT or MHC‐II KO bone marrow (BM) and adoptively transferred with CD90.1^+^ WT CD4^+^ T cells (Fig. 2A). We found that the CD90.1^+^ Tregs adoptively transferred into MHC‐II KO BM chimeric mice were unable to maintain their numbers in the peripheral blood to the same extent as WT BM chimeric mice 20 days post‐transfer (Fig. 2B). Linked to this, CD90.1^+^ Tconvs in the MHC‐II KO BM chimeric mice expressed significantly lower IL‐2 mRNA in comparison to Tconvs from WT BM chimeric mice (Fig. 2C). Furthermore, we utilized IL‐2/anti IL‐2 antibody immune complexes (IL‐2 IC) to determine if the lack of IL‐2 was playing a role in the maintenance of Tregs in this model 11. Indeed, we found that treatment with IL‐2 IC partially restored Treg percentages in the peripheral blood of the MHC‐II KO BM chimeric mice, suggesting that the lack of IL‐2 contributes to the failure to maintain Tregs in the absence of MHC‐II/TCR interactions (Fig. 2B).

**Figure 3:**
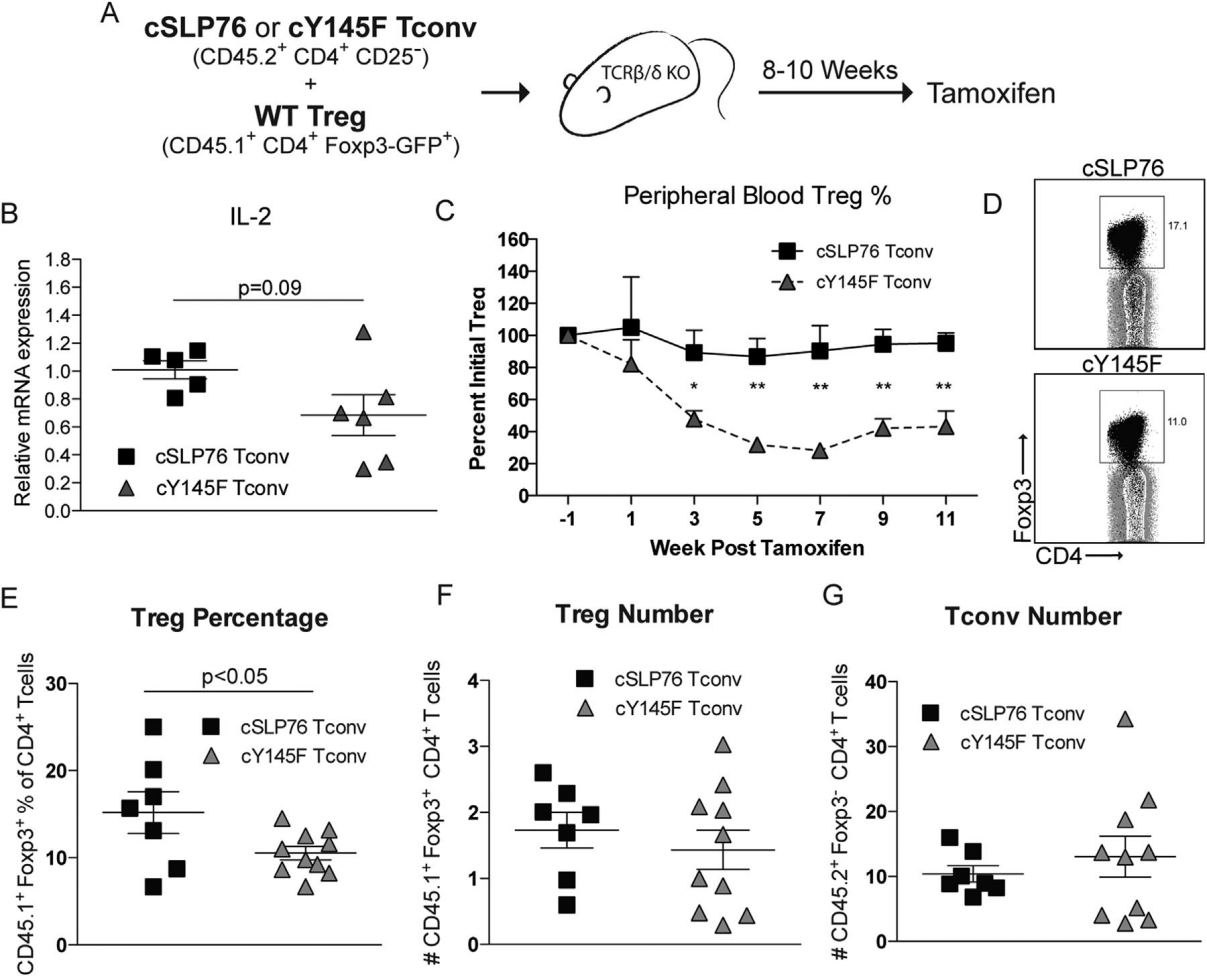
Selective ablation of TCR signaling in CD4^+^ Tconvs leads to decreased Treg numbers (A). FACS‐sorted CD45.2^+^ Tconvs (CD4^+^CD25^−^) from conditional WT SLP‐76 (cSLP76) or conditional Y145F mutant SLP‐76 (cY145F) mice were mixed with CD45.1^+^ WT Tregs and transferred into TCR β/δ KO mice at a 4:1 ratio. After 8–10 weeks, the mice were treated with Tamoxifen. (B) IL‐2 mRNA was assessed in FACS‐sorted splenic CD45.2^+^CD4^+^ T cells at week 5 post‐Tamoxifen treatment. Summary data from two independent experiments are represented by mean ± SEM with *n* = 5–6/group. (C) The change in percentage of peripheral blood CD45.1^+^CD4^+^Foxp3^+^ Tregs was plotted against time post‐Tamoxifen treatment. One representative experiment is plotted as the mean ± SEM (*n* = 4–6/group) of three independent experiments. (D) Representative flow plots of total CD4^+^ T cells from mouse spleens at 12 weeks post‐Tamoxifen treatment is shown. The number in each plot represents the proportion of CD45.1^+^CD4^+^Foxp3^+^ cells of total CD4^+^ T cells. (E) A summary graph of two independent experiments depicts mean percentage CD45.1^+^CD4^+^Foxp3^+^ cells of total CD4^+^ T cells in the spleen at week 12 ± SEM of *n* = 7–10/group. (F) A summary graph of two independent experiments depicting mean number of CD45.1^+^CD4^+^Foxp3^+^ T cells in the spleen at week 12 ± SEM of *n* = 7–10/group. G. A summary graph of two independent experiments depicting mean number of CD45.2^+^CD4^+^Foxp3^−^ T cells in the spleen at week 12 ± SEM of *n* = 7–10/group. *p < 0.05 and **p < 0.01 or as noted by unpaired, two tailed Student's *t*‐test.

In MHC‐II KO BM chimeric mice, Tregs and Tconvs both lose MHC‐II/TCR interactions. Thus, the lack of TCR signaling by Tregs could also contribute to defective maintenance in this model. In order to more fully test whether the baseline TCR signaling ability of Tconvs alone was important in the maintenance of Tregs in vivo, it was necessary to utilize a second system whereby TCR signaling was attenuated in Tconvs but not in Tregs. To accomplish this task, we designed an adoptive transfer model in which TCR signals could be inducibly decreased specifically in Tconvs (Fig. 3A). We utilized a Tamoxifen‐inducible system in which a WT loxp‐flanked SLP‐76 allele is deleted upon treatment, leaving either a single WT SLP‐76 allele (cSLP76) or a SLP‐76 Y145F mutant allele (cY145F) 12. Tconv from either cSLP76 or cY145F mice were mixed with WT Tregs at a 4:1 ratio and adoptively transferred into T cell‐deficient TCRβ/δ KO mice. We waited for T cell reconstitution and steady state to be reached, for example, until the peripheral blood CD4^+^ T cells reached a constant percentage of lymphocytes (∼8–10 weeks). The mice were then treated with Tamoxifen to induce deletion of the loxp‐flanked WT SLP‐76 allele (Fig. 3A). Five weeks after Tamoxifen treatment, Tconvs from peripheral lymphoid organs were analyzed for IL‐2 expression. The CD45.2^+^CD4^+^ Tconvs from the spleen of the cY145F adoptive transfer showed a trend toward decreased IL‐2 mRNA expression in comparison to the Tconvs from the cSLP76 transfer (Fig. 3B). Longitudinal analysis of the Treg percentage (of CD4^+^ T cells) in the peripheral blood showed a significant decrease in mice transferred with cY145F compared to cSLP76 Tconvs starting at week 3, which was sustained through week 12 (Fig. 3C). Moreover, at week 12 post‐Tamoxifen treatment, we found that CD45.1^+^ WT Tregs made up a smaller fraction of the total CD4^+^ T cell pool in the spleens of mice with Tconvs from cY145F compared to cSLP76 mice (Fig. 3D and E). This was observed despite finding variable reconstitution levels of Tconvs between mice within each group, further suggesting that the primary effect of this manipulation in our model was the ability of Treg numbers to be indexed to the TCR signaling capacity of Tconvs (Fig. 3F and G). Together, these data suggest that TCR signaling by Tconvs is important for steady state IL‐2 production, which correlates with their ability to maintain Tregs.

Our data provided here in this manuscript support a model by which Treg numbers are indexed to IL‐2 produced by Tconvs through TCR stimulation by self peptide/MHC‐II complexes. We examined IL‐2 production from Tconvs in vivo using quantitative PCR, since direct measurement of IL‐2 protein production in a naive mouse is difficult to measure, as steady state IL‐2 most likely remains local to the secondary lymphoid organs. Still, antibody neutralization of IL‐2 demonstrates that a functionally significant amount of IL‐2 plays a role in maintenance of peripheral Tregs 4. Using CD5 as a measure of TCR affinity for self peptide/MHC‐II complexes showed that high TCR affinity of Tconvs correlated with increased IL‐2 mRNA expression. Moreover, an acute decrease in TCR signaling by Tconvs through the Y145F mutation showed a trend toward decreased IL‐2 mRNA expression. The latter analysis may not have reached statistical significance due to the nature of the Tamoxifen‐inducible system, which results in incomplete deletion of the loxp‐flanked WT SLP‐76 allele. Thus, the contamination of Tconvs that have not deleted SLP‐76 may have contributed to higher IL‐2 mRNA expression in this setting. Further support of this concept was found in the observation of significantly decreased IL‐2 mRNA in Tconvs transferred into an MHC‐II deficient environment. This was associated with a decrease in Treg proportions that was partially rescued after treatment with IL‐2 IC.

The ability of Tconvs to produce IL‐2 in response to TCR stimulation has long been appreciated as a result of signals provided by an activating, often foreign, antigen. The potential for Tconvs to produce IL‐2 at baseline, or from non‐activating ligands has not been studied despite evidence for its existence, primarily from observations of normal percentages of Tregs in germ‐free mice 13, 14. The absence of foreign antigen in germ‐free mice suggests that the IL‐2 required for Treg maintenance is not produced through stimulation of the TCR on Tconvs by foreign peptides presented on MHC‐II. Therefore, we propose that baseline IL‐2 production is a result of TCR interactions with self‐peptide MHC‐II complexes.

Tregs are characterized to be part of either central or effector subsets, which are distinguished by varying surface receptor phenotypes, proliferative capacity, function, and dependence on IL‐2 15, 16, 17. Given that IL‐2 was dependent on TCR signaling by Tconvs, one would predict that the Tregs remaining in the cY145F adoptive transfer would be enriched for the IL‐2‐independent and highly proliferative effector Treg subset. However, we could not test this in our adoptive transfer model, as in all locations observed (spleen, LN, gut, mLN) the WT Treg had an effector phenotype, regardless of whether the adoptively transferred Tconv were of cSLP76 and cY145F origin (data not shown). This phenotype was likely acquired during the lymphopenic expansion that occurred prior to the SLP‐76 deletion, which precluded the analysis of the Treg subsets that remained.

Despite the caveat of initial lymphopenic expansion for reconstitution of the T cell‐deficient mouse, our adoptive transfer model corrected for a number of concerns involved in testing the role of TCR signaling by Tconvs in Treg maintenance. First, the SLP‐76 protein was altered only in Tconvs, allowing for all Treg‐intrinsic homeostatic factors to remain intact. Second, deletion of SLP‐76 using the Tamoxifen‐inducible cre‐lox system negated the concern that Tconvs with decreased TCR signaling would develop differently than the WT controls, potentially leading to an altered TCR repertoire or cytokine production downstream of TCR stimulation. Finally, in order to minimize any confounding factors associated with lymphopenic expansion, we deleted the loxp‐flanked SLP‐76 allele only after the transferred cells of both types (cSLP76 and cY145F) fully expanded and reached a steady state.

### Concluding Remarks

The results of these studies demonstrate a previously unrecognized role of TCR affinity of Tconvs to self‐peptide MHC‐II complexes in the maintenance of Tregs. These are novel findings because they link the size of the Treg population not only to activated T cells producing high levels of IL‐2, but to the broad level of self‐reactivity found in the Tconv pool. It is reasonable then to propose that thymic positive selection of CD4^+^ Tconvs, which creates a population of Tconvs with low affinity to self‐peptide MHC‐II complexes, allows Tregs to be appropriately indexed to Tconvs in the steady state for immune tolerance. Overall, these observations are important in the understanding of autoimmunity because they demonstrate a potential avenue in which self‐tolerance mechanisms can fail. Additionally, they provide important considerations in formulating Treg‐based immunotherapies, as the transfer of Tregs paired with inhibition of TCR signaling might actually lessen the ability of the transferred Tregs to maintain sufficient numbers to be effective over time.

## Conflicts of Interest

The authors declare no conflicts of interest.

## References

[1] Sakaguchi, S. , N. Sakaguchi , M. Asano , M. Itoh , and M. Toda . 1995 Immunologic self‐tolerance maintained by activated T cells expressing IL‐2 receptor alpha‐chains (CD25). Breakdown of a single mechanism of self‐tolerance causes various autoimmune diseases. J. Immunol. 155:1151–1164. 7636184

[2] Fontenot, J. D. , M. A. Gavin , and A. Y. Rudensky . 2003 Foxp3 programs the development and function of CD4+CD25+ regulatory T cells. Nat. Immunol. 4:330–336. DOI:10.1038/ni904 12612578 10.1038/ni904

[3] Fisson, S. , G. Darrasse‐Jeze , E. Litvinova , F. Septier , D. Klatzmann , R. Liblau , and B. L. Salomon . 2003 Continuous Activation of Autoreactive CD4+ CD25+ Regulatory T Cells in the Steady State. J. Exp. Med. 198:737–746. DOI:10.1084/jem.20030686 12939344 10.1084/jem.20030686 PMC2194185

[4] Setoguchi, R. 2005 Homeostatic maintenance of natural Foxp3+ CD25+ CD4+ regulatory T cells by interleukin (IL)‐2 and induction of autoimmune disease by IL‐2 neutralization. J. Exp. Med. 201:723–735. DOI:10.1084/jem.20041982 15753206 10.1084/jem.20041982 PMC2212841

[5] Bhandoola, A. , X. Tai , M. Eckhaus , H. Auchincloss , K. Mason , S. A. Rubin , K. M. Carbone , Z. Grossman , A. S. Rosenberg , and A. Singer . 2002 Peripheral expression of self‐MHC‐II influences the reactivity and self‐tolerance of mature CD4(+) T cells: evidence from a lymphopenic T cell model. Immunity 17:425–436. 12387737 10.1016/s1074-7613(02)00417-x

[6] Gavin, M. A. , S. R. Clarke , E. Negrou , A. Gallegos , and A. Rudensky . 2002 Homeostasis and anergy of CD4(+)CD25(+) suppressor T cells in vivo. Nat. Immunol. 3:33–41. DOI:10.1038/ni743 11740498 10.1038/ni743

[7] Almeida, A. R. M. , B. Zaragoza , and A. A. Freitas . 2006 Indexation as a novel mechanism of lymphocyte homeostasis: the number of CD4+CD25+ regulatory T cells is indexed to the number of IL‐2‐producing cells. J. Immunol. 177:192–200. 16785514 10.4049/jimmunol.177.1.192

[8] Jordan, M. S. , J. E. Smith , J. C. Burns , J.‐E. T. Austin , K. E. Nichols , A. C. Aschenbrenner , and G. A. Koretzky . 2008 Complementation in trans of altered thymocyte development in mice expressing mutant forms of the adaptor molecule SLP76. Immunity 28:359–369. DOI:10.1016/j.immuni.2008.01.010 18342008 10.1016/j.immuni.2008.01.010 PMC2323515

[9] Azzam, H. S. , A. Grinberg , K. Lui , H. Shen , E. W. Shores , and P. E. Love . 1998 CD5 expression is developmentally regulated by T cell receptor (TCR) signals and TCR avidity. J. Exp. Med. 188:2301–2311. 9858516 10.1084/jem.188.12.2301 PMC2212429

[10] Mandl, J. N. , J. P. Monteiro , N. Vrisekoop , and R. N. Germain . 2012 T cell‐positive selection uses self‐ligand binding strength to optimize repertoire recognition of foreign antigens. Immunity 38:263–274. DOI:10.1016/j.immuni.2012.09.011 23290521 10.1016/j.immuni.2012.09.011 PMC3785078

[11] Webster, K. E. , S. Walters , R. E. Kohler , T. Mrkvan , O. Boyman , C. D. Surh , S. T. Grey , and J. Sprent . 2009 In vivo expansion of T reg cells with IL‐2‐mAb complexes: induction of resistance to EAE and long‐term acceptance of islet allografts without immunosuppression. J. Exp. Med. 206:751–760. DOI:10.1126/science.180605 19332874 10.1084/jem.20082824 PMC2715127

[12] Wu, G. F. , E. Corbo , M. Schmidt , J. E. Smith‐Garvin , M. J. Riese , M. S. Jordan , T. M. Laufer , E. J. Brown , and J. S. Maltzman . 2011 Conditional deletion of SLP‐76 in mature T cells abrogates peripheral immune responses. Eur. J. Immunol. 41:2064–2073. DOI:10.1002/eji.201040809 21469089 10.1002/eji.201040809 PMC3124603

[13] Min, B. , A. Thornton , S. M. Caucheteux , S.‐A. Younes , K. Oh , J. Hu‐Li , and W. E. Paul . 2007 Gut flora antigens are not important in the maintenance of regulatory T cell heterogeneity and homeostasis. Eur. J. Immunol. 37:1916–1923. DOI:10.1002/eji.200737236 17549737 10.1002/eji.200737236

[14] Östman, S. , C. Rask , A. E. Wold , S. Hultkrantz , and E. Telemo . 2006 Impaired regulatory T cell function in germ‐free mice. Eur. J. Immunol. 36:2336–2346. DOI:10.1002/eji.200535244 16897813 10.1002/eji.200535244

[15] Smigiel, K. S. , E. Richards , S. Srivastava , K. R. Thomas , J. C. Dudda , K. D. Klonowski , and D. J. Campbell . 2014 CCR7 provides localized access to IL‐2 and defines homeostatically distinct regulatory T cell subsets. J. Exp. Med. 211:121–136. DOI:10.4049/jimmunol.1200507 24378538 10.1084/jem.20131142 PMC3892972

[16] Campbell, D. J. , and M. A. Koch . 2011 Phenotypical and functional specialization of FO XP3. Nat Rev Immunol. 11:119–130. DOI:10.1038/nri2916 21267013 10.1038/nri2916 PMC3289970

[17] Siegmund, K. 2005 Migration matters: regulatory T‐cell compartmentalization determines suppressive activity in vivo. Blood 106:3097–3104. DOI:10.1182/blood‐2005‐05‐1864 16014565 10.1182/blood-2005-05-1864 PMC1895340

